# Gastrocnemius Release in the Management of Chronic Plantar Fasciitis: A Systematic Review

**DOI:** 10.1177/10711007211052290

**Published:** 2021-11-12

**Authors:** Zaki Arshad, Aiman Aslam, Mohammad A. Razzaq, Maneesh Bhatia

**Affiliations:** 1 School of Clinical Medicine, University of Cambridge, Cambridge, UK; 2 Department of Trauma and Orthopaedic Surgery, University Hospitals of Leicester NHS Trust, Leicester, UK

**Keywords:** plantar fasciitis, gastrocnemius recession, gastrocnemius lengthening, systematic review

## Abstract

**Background:**

This systematic review aims to summarize the outcomes of gastrocnemius recession in the treatment of plantar fasciitis.

**Methods:**

A systematic review was performed according to PRISMA guidelines using the PubMed, Embase, Emcare, Web of Science, Scopus, and CINAHL databases. A 2-stage title/abstract and full text screening process was performed independently by 2 reviewers. Randomized controlled trials, cohort, and case-control studies reporting the results of gastrocnemius recession in patients with plantar fasciitis were included. The MINORS and Joanna Briggs Institute Criteria were used to assess study quality and risk of bias.

**Results:**

A total of 285 articles were identified, with 6 of these studies comprising 118 patients being ultimately included. Significant postoperative improvement in American Orthopaedic Foot & Ankle Society, visual analog scale, 36-Item Short Form Health Survey, Foot Forum Index, and Foot and Ankle Ability Measure scores were reported. Included studies also described an increase in ankle dorsiflexion range of motion and plantarflexion power. An overall pooled complication rate of 8.5% was seen, with persistent postoperative pain accounting for the most common reported complication. Gastrocnemius recession is associated with greater postoperative improvement than plantar fasciotomy and conservative stretching exercises.

**Conclusion:**

The current evidence demonstrates that gastrocnemius recession is effective in the management of plantar fasciitis, specifically in patients with gastrocnemius contracture who do not respond to conservative treatment.

**Level of Evidence:**

Level III, Systematic review of level I-III studies.

## Introduction

Plantar fasciitis refers to degeneration and inflammation of the proximal plantar fascia.^15,25^ The condition presents with plantarmedial heel pain, often exacerbated following periods of inactivity, such as upon waking in the morning.^15,21^ Approximately 1 million adults receive treatment for plantar fasciitis in the United States of America alone every year, with around 1/10th of all adults being affected at some point during their life.^26,32^

A number of conservative treatment options are available, including nonsteroidal anti-inflammatory drugs (NSAIDs), physical therapy, corticosteroid injections, and orthoses.^15,21,24^ Such conservative regimes provide symptom resolution within 12 months in approximately 90% of cases.^21,26,35^ Should symptoms persist following conservative measures, operative techniques such as plantar fasciotomy can be employed.^5,15,16,21^

Although a number of risk factors have been proposed, the pathophysiology of plantar fasciitis is not well understood.^21^ One emerging area of interest is that of the role of gastrocnemius tightness. There is a close anatomical, functional, mechanical, and histologic relationship between the Achilles tendon and the plantar fascia and numerous authors have demonstrated an association between gastrocnemius contracture and plantar fasciitis.^2,14,25,28,29,34^ It is postulated that contracture of the gastrocnemius increases Achilles tendon tension and limits ankle dorsiflexion.^12,21,25,28,29^ This may interfere with the windlass mechanism and lead to increased strain on the plantar fascia and calcaneal tuberosity.^12,21,31^ The recent study by Pearce et al^30^ provides further evidence in favor of this theory by demonstrating a strong significant correlation between gastrocnemius tightness and heel pain severity in plantar fasciitis.

It therefore follows that release of the gastrocnemius muscle initially through stretching exercises and, in recalcitrant cases, through operative recession/lengthening, may aid in the management of plantar fasciitis.^4,7,10,19^ Previous studies have demonstrated good outcomes following operative gastrocnemius recession.^1,4,19^ However, despite an increase in popularity, there appears to be a lack of widespread consensus regarding the use of gastrocnemius recession in chronic plantar fasciitis, with a number of alternative treatment options available.^3,6,9,12^ Furthermore, there is currently no other systematic review specifically investigating the use of gastrocnemius recession in recalcitrant plantar fasciitis.

Thus, this review aims to address this issue by systematically summarizing the current literature with respect to outcomes and complications reported following the use of gastrocnemius recession for chronic plantar fasciitis. In doing so, we hope to better inform clinicians regarding the effectiveness of the procedure and answer the question of whether its use should be advocated in patients suffering from chronic plantar fasciitis.

## Methods

### Search Strategy

A systematic electronic search was performed by 2 reviewers independently using PubMed, Medline, Embase, Emcare, ISI Web of Science, and Scopus. The final search strategy was produced by combining relevant terms such as *plantar fasciitis*, *plantar fasciopathy*, *heel spur syndrome*, and *gastrocnemius*, with the Boolean operators (*and*, *or*). The title of the systematic review was registered in the Open Science Framework. All aspects of the Preferred Reporting Items for Systematic reviews and Meta-Analysis (PRISMA) guidelines were followed while performing the systematic review.^18^ The individual study inclusion and exclusion criteria were established a priori. Original research studies with a level of evidence of III or higher (case control, cohort, randomized controlled trials) evaluating the results of gastrocnemius recession in human patients with chronic plantar fasciitis were included. No specific control group was required for inclusion. Retrospective case series articles, conference abstracts, review articles, commentaries and case reports were excluded. No restriction on date of publication was imposed. Only studies evaluating the results of gastrocnemius recession alone, with no concomitant procedures, were included. Studies with additional comparison groups were included provided it was possible to clearly separate data pertaining to gastrocnemius recession.

### Data Management

Studies were imported into the Rayyan web-based reference management tool (http://rayyan.qcri.org) to aid screening and selection.^27^

### Selection Process

Two reviewers independently performed a 2-stage title/abstract and full-text screening to identify eligible studies. Differences in opinion at any stage were resolved by discussion. A third reviewer was consulted in the event of a discrepancy or if no consensus was reached.

### Data Extraction

An extraction spreadsheet was created in Microsoft Excel with headings as follows: (1) Author, (2) Year of publication, (3) Title, (4) Number of patients and feet, (5) Age, (6) Male: female sex ratio, (7) Presence of gastrocnemius contracture, (8) Previous treatment, (9) Outcomes, (10) Follow-up period, and (11) Complications. This spreadsheet was used by 2 authors to extract information from all studies.

### Data Synthesis

**Figure 1.**
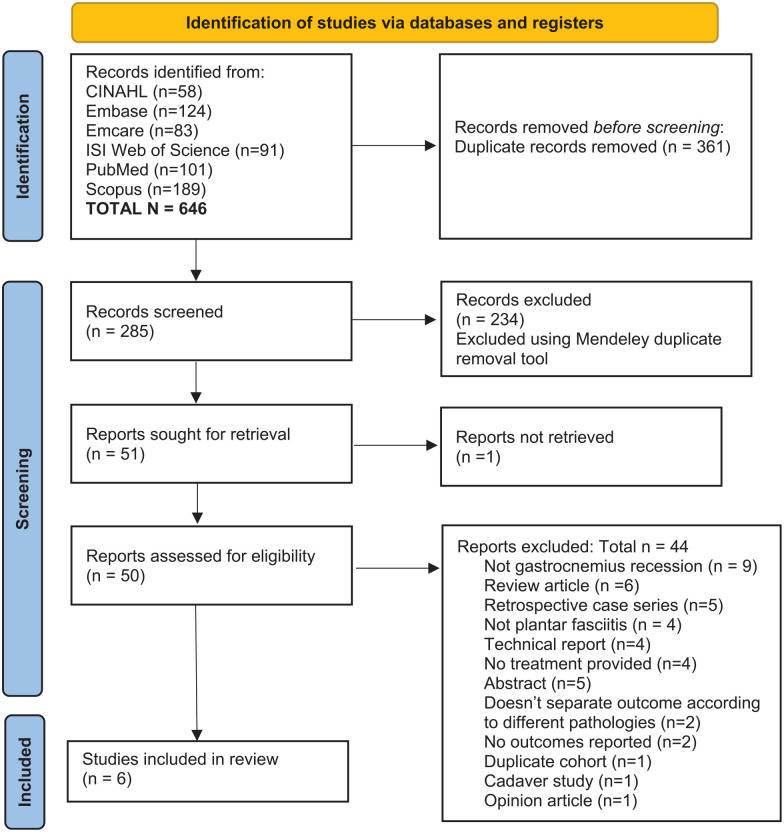
PRISMA flow diagram showing the number of studies retrieved and excluded at both screening stages.

Results of the search and screening processes are displayed in the PRISMA flow diagram (Figure 1). A qualitative thematic synthesis approach is taken, with results reported in separate sections focusing on outcomes such as outcome scale scores, range of motion, gastrocnemius strength, and complications.

### Risk of Bias and Quality of Evidence Assessment

The Methodological Index for Non Randomized Studies (MINORS) criteria were used to assess the risk of bias and quality of all included case series and cohort studies.^33^ The MINORS criteria comprises a 12-item checklist, each item given a score of 0 (not reported), 1 (inadequately reported), or 2 (adequately reported). The studies were scored against a maximum of 16 points for noncomparative studies and 24 points for comparative studies. The quality of randomized controlled trials was assessed using the Joanna Briggs Institute critical appraisal checklist. This consists of a 13-item checklist, with each item scored using either “yes,” “no,” or “not reported.”^23^

## Results

A total of 285 unique studies were identified and screened, of which 6 studies (2.1%) comprising a total of 118 patients (123 feet) undergoing gastrocnemius release for plantar fasciitis were included (Figure 1). Two Level I randomized controlled trials, 3 Level II cohort studies, and 1 Level III case control study were included (Table 1). Results of the quality assessment process are detailed in the Supplementary Material S1 and S2.

**Table 1.** Summary of Included Studies.

Author	Year	Study Type	Number of Patients	M:F	Mean Age, y (range)	Mean Follow-up, mo (range)	Symptom Duration
Chimera et al^4^	2010	Case-control	4 (7 feet)	6:1	50.6	3	NR; all received conservative treatment
Gamba et al^9^	2020	RCT	17	2:15	46.2	12	>9 mo; all received conservative treatment
Hoefnagels et al^12^	2020	Cohort	32	50	9:23	12	All patients received at least 3 different conservative treatments in addition to 8 wk of plantar fascia and Achilles tendon stretching prior to surgery
Huang et al^13^	2018	Cohort	15 (17 feet)	9:6	47.0	12	>6 mo of conservative treatment
Molund et al^19^	2018	RCT	20	5:15	46 (29-68)	12	>12 mo; all received conservative treatment
Monteagudo et al^22^	2013	Cohort	30	16:14	44 (21-63)	12	14 (10-64); all received conservative treatment

Abbreviations: M:F, male-to-female ratio; NR, not recorded; RCT, randomized controlled trial.

### Outcomes

Study outcomes with respect to posttreatment changes in outcomes scores are detailed in Table 2.

**Table 2.** Treatment and Pre- and Posttreatment Scores of Studies.

Author	Treatment	Mean Pretreatment Score	Mean Posttreatment Score	Significant Improvement?
Chimera et al^4^	Gastrocnemius recession (Strayer technique distal to musculotendinous junction)	FAAM: 59	FAAM: 91	Significant postoperative improvement (*P* = .016); postoperative FAAM for surgical group significantly lower than in healthy controls (*P* = .016)
Gamba et al^9^	Proximal medial gastrocnemius recession	AOFAS: 65.3±10.4 VAS: 68.1±18.8 SF-36: 35.2±9.2	AOFAS: 89±9.9 VAS: 15.1±18.3 SF-36: 43.8±12.7	Both groups showed significant postoperative improvement in SF-36 (*P* = .01 and *P* = .00), AOFAS, and VAS scores (no *P* values given); no significant differences in postoperative scores between the 2 treatment groups (*P* = .24 for AOFAS, *P* = .14 for VAS, *P* = .75 for VAS)
	Plantar fasciotomy	AOFAS: 68.7±8.2 VAS: 69.5±18 SF-36: 34.7±10.7	AOFAS: 86.7±12.1 VAS: 28.7±25.6 SF-36: 46.4±10.5	
Hoefnagels et al^12^	Gastrocnemius recession (Gastrocslide procedure 15-20 cm above the medial malleolus)	VAS: 78±19	VAS: 20±24	FFIB, FFIC, FAAM, FAAM sports, VAS, and VISA-A scores improved significantly postoperatively (all *P* < .001); however, mean values were only reported for VAS
Huang et al^13^	Gastrocnemius recession (either proximal recession or endoscopic distal technique at level of musculotendinous junction)	VAS: 6.86±1.57 AOFAS: 39.14±15.91 SFMCS: 76.34±10.45	VAS: 1.57±2.30 AOFAS: 87.00±12.95 SFMCS: 78.99±12.34	Significant differences between preoperative and postoperative scores not tested; mean SFMCS score significantly higher in those who underwent both procedures concurrently compared to recession alone (*P* < .05), but not when compared to microtenotomy alone (*P* = .08); no significant differences in any other outcome scores used
	Radiofrequency microtenotomy	VAS: 7.21±1.69 AOFAS: 42.0±13.47 SFMCS: 71.24±15.06	VAS: 1.50±2.43 AOFAS: 88.54±16.79 SFMCS:83.45±11.49	
	Gastrocnemius recession + radiofrequency microtenotomy	VAS: 6.68±1.77 AOFAS: 50.56±18.75 SFMCS: 67.95±29.57	VAS: 1.29±2.22 AOFAS: 90.71±13.51 SFMCS: 94.00±5.21	
Molund et al^19^	Proximal medial gastrocnemius recession	AOFAS: 59.5 (42-76) VAS: 7.6 (3.9-10) SF-36: 65 (40-95)	AOFAS: 88 (55-100) VAS: 2.8 (0-8.1) SF-36: 90 (55-100)	Significant postoperative increases in all scores in the operative group (all *P* < .001); no significant increases in the conservative group; operative group had significantly higher 12-mo outcome scores than the nonoperative group (AOFAS: *P* < .001, VAS: *P* = .001, SF-36: *P* = .007)
	Conservative stretching	AOFAS: 52.5 (37-73) VAS: 7.6 (3.9-10) SF-36: 55 (25-95)	AOFAS: 65.5 (31-88) VAS: 7.4 (0.2-9.3) SF-36: 63 (15-100)	
Monteagudo et al^22^	Proximal medial gastrocnemius Recession	VAS: 8.2 AOFAS: 46	VAS: 0.9 AOFAS: 90	Significant differences not determined
	Plantar Fasciotomy	VAS: 8.1 AOFAS: 48	VAS: 3.1 AOFAS: 66	

Abbreviations: AOFAS, American Orthopaedic Foot & Ankle Society score; FAAM, Foot and Ankle Ability Measure; FFIB: Foot function index B; FFIC: Foot function index C; SF-36, 36-Item Short Form Health Survey; SFMCS, Short-Form (36) Mean Component Score; VAS, visual analog scale. VISA-A: Victorian Institute of Sport Assessment-Achilles.

**Table 3.** Summary of Postoperative Complications Described in Included Studies.

Study	Number of Feet	Nervous System	Wound Healing	Other	Total, n/N (%)
Chimera et al^4^	7	–	–	–	0
Gamba et al^9^	17	1 sural nerve lesion	1 superficial wound infection	–	2/17 (11.8)
Hoefnagels et al^12^	32	1 sural nerve neuropraxia	1 superficial wound infection	1 complex regional pain syndrome	3/32 (9.4)
Molund et al 2018^19^	20	–	–	3 persistent swelling/pain, 2 of which resolved within 1 y	5/20 (15)
Monteagudo et al^22^	30	–	–	1 calf hematoma	1/30 (3.3)
Total, n (%)	106	2/106 (1.9)	2/106 (1.9)	5/106 (4.7)	9/106 (8.5)

Gastrocnemius recession was performed at a variety of different levels (Table 2). A total of 3 studies performed a proximal medial recession, 1 study used a Strayer approach, 1 a gastrocslide procedure 15 to 20 cm above the medial malleolus and one study used either a proximal medial recession or an endoscopic technique at the level of the musculotendinous junction. Unfortunately, it was not possible to compare outcomes according to the exact level of gastrocnemius recession because of the small number of studies using each specific approach and heterogeneity in outcome measures used.

### Postoperative Regime

The postoperative protocol was reported by 5 included studies. All studies describe patients being allowed to weightbear as tolerated; however, differences are noted in the additional immobilization techniques. A postoperative boot is described in one study (Chimera et al^4^). Hoefnagels et al^12^ describes use of a plaster cast for 2 weeks, followed by 4 weeks night splint, whereas Monteagudo et al^22^ and Gamba et al^9^ use a rigid open shoe for the first 2 postoperative weeks. No casts, boots, or rigid shoes are used postoperatively by Molund et al.^19^ Unfortunately, it was again not possible to effectively evaluate the impact of different postoperative regimes on outcomes because of the large heterogeneity in outcome measures used across these different studies.

### Range of Motion

Range of motion outcomes were reported by a total of 3 studies: Hoefnagels et al,^12^ Molund et al,^19^ and Chimera et al^4^ reported significant postoperative increases in ankle dorsiflexion with the knee in full extension.

### Return to Walking, Work, and Sports

The study of Monteagudo et al^21^ reports that comfortable weightbearing was achieved after 1 week in the gastrocnemius recession group, compared with >4 weeks in the plantar fasciotomy group. The gastrocnemius recession group also showed a decreased mean return to work of 3 weeks (range, 1-12) compared with 12 weeks in the fasciotomy group. A similar effect was seen with respect to return to sports, with a mean time of 5 weeks in the gastrocnemius recession group and 16 weeks in the fasciotomy group.

### Gastrocnemius Strength

Hoefnagels et al^12^ reported that all patients were able to perform 20 bilateral and 5 unilateral heel raises 1 year postoperatively. Calf power was assessed using 10 consecutive single heel rises in the study of Gamba et al,^9^ with no patient in either the gastrocnemius recession or plantar fasciotomy group demonstrating reduced power at final follow-up. Chimera et al^4^ is the only study to test isometric and isokinetic ankle plantarflexion torque. Peak isokinetic plantarflexion strength significantly increased 3 months postoperatively. However, the results of this study should be interpreted with caution because of the small cohort size and case-control study design. The randomized controlled trial of Molund et al^19^ used an Achilles test battery to evaluate the performance of the Achilles muscle-tendon complex. A significant postoperative increase in toe-raise endurance and a decrease in countermovement jump height was observed. No significant differences were seen when comparing those receiving gastrocnemius recession and nonoperative treatment

### Regression Analysis

Only a single study, Huang et al,^13^ performed regression analysis to identify predictors of postoperative outcomes. Linear regression showed no significant association between patient age, gender, height, weight, body mass index, bilateral vs unilateral procedure, type of intervention (gastrocnemius recession, radiofrequency microtenotomy, or both), baseline visual analog scale (VAS), American Orthopaedic Foot & Ankle Society score (AOFAS), or 36-Item Short Form Health Survey (SF-36) and postoperative VAS, AOFAS, or SF-36 scores. Furthermore, binary logistic regression showed that none of these preoperative variables were able to predict patient satisfaction or meeting of expectations.

### Complications

A total of 5 studies containing 106 feet undergoing gastrocnemius recession report complications were associated with this intervention (Table 3). An overall pooled complication rate of 9 of 106 (8.5%) is seen across these patients, with the most common complication being persistent swelling/pain that resolved withing 1 year.

## Discussion

This review aimed to systematically identify and summarize current literature pertaining to the outcomes associated with gastrocnemius release in patients with chronic plantar fasciitis. A total of 6 studies were included, with 2 of these being high-quality randomized control trials, 3 cohort studies, and 1 case control study. Results of the critical appraisal process using the MINORS criteria show that the quality of included studies was generally good. One flaw seen across all studies was a lack of description as to blind assessment of subjective and objective study outcomes, which may provide some bias in outcome measurement. Furthermore, owing to heterogeneity in study design, it was not possible to perform a meta-analysis comparing different treatment options such as gastrocnemius recession alone, recession with concomitant procedures, and plantar fasciotomy.

All studies report excellent outcomes associated with the use of gastrocnemius release for chronic plantar fasciitis. Significant postoperative improvements are described via a variety of outcome assessment scales, including AOFAS, VAS, SF-36, and FAAM. Comparison between gastrocnemius recession and other treatments are described by 4 studies. Gamba et al^9^ find no significant differences in outcome between gastrocnemius release and plantar fasciotomy. Although Monteagudo et al^21^ did not provide the results of any formal statistical comparison between these 2 treatments, a “profound and long-lasting effect” on VAS and AOFAS scores has been reported. Furthermore, on inspection of this study’s results, a greater improvement in AOFAS and VAS scores is clearly seen in the gastrocnemius recession group compared to the plantar fasciotomy group. This combined with an increased patient satisfaction, quicker time to improvement, weightbearing, return to work and sports, and lower complication rate have led the authors to conclude “conventional PPF (partial proximal fasciotomy) compares poorly to PMGR (proximal medial gastrocnemius release) in terms of success and patient satisfaction.” Furthermore, Molund et al^19^ find significantly higher 12-month postoperative AOFAS, VAS, and SF-36 scores in those receiving gastrocnemius recession compared with patients treated with conservative stretching exercises. These comparative studies therefore largely provide evidence favoring the use of gastrocnemius recession over other treatments such as plantar fasciotomy and conservative therapy. Huang et al^13^ describe a significantly greater improvement in SFMCS in those undergoing gastrocnemius recession with concurrent microtenotomy compared with either procedure alone, with no significant difference reported between the results of either procedure when performed alone. However, further research is required into the effects of gastrocnemius release combined with other procedures such as plantar fasciotomy or microtenotomy before firm conclusions may be drawn.

The potential benefits of gastrocnemius release are further demonstrated through included studies reporting significant postoperative increases in ankle dorsiflexion range of motion and plantarflexion torque.^4,12,19^ Furthermore, most patients maintain an ability to perform single calf raises.^12^ These results are important as one traditional concern regarding the use of gastrocnemius recession is loss of gastrocnemius power, which does not appear to be the case.

Across the 5 studies reporting postoperative complications, an overall complication rate of 8.5% is seen. Further breakdown of these complications reveals that a number of these are relatively minor complications that may be associated with any operative procedure, such as wound infection. Nerve injury—particularly to the sural nerve—is relatively rare, occurring in 1.9% of patients. Nevertheless, surgeons should therefore be aware of this complication, particularly when using a distal recession technique, and take extreme care to identify and avoid damage to nervous structures.

Of the 6 included studies, all except Gamba et al^9^ report that patients were only included if they were shown to be suffering from gastrocnemius contracture. Although the results of this review show that gastrocnemius recession is effective in these patients, it is not clear what proportion of patients with plantar fasciitis suffer from underlying gastrocnemius contracture. The favored technique for diagnosing gastrocnemius contracture is the Silfverskiöld test.^8,12,13,17,22^ However, this test is not without controversy, with debate existing as to the best way to perform it and what constitutes a positive result.^11,17^ A recent study advocates instead for the use of a new range of motion measuring device that may show greater reliability.^20,29^ Further research in evaluating testing methods is required to facilitate the accurate and identification of patients with gastrocnemius contracture, who may benefit from a recession procedure.

It is not known to what extent the presence of a gastrocnemius contracture may influence the outcome of gastrocnemius recession. Huang et al^13^ is the only study to attempt to identify patient- and treatment-related factors that may affect outcomes, failing to identify any prognostic factors. However, it is not appropriate to draw any firm conclusions regarding prognostic factors from a single 15-patient study. Further high-quality larger cohort studies are certainly required.

Furthermore, most included studies report that patients received conservative treatment before undergoing operative intervention, and previous studies suggest that this may benefit up to 90% of patients.^21^ However, there is currently no literature investigating the role of patient- and treatment-related factors in predisposing specific patients to a good or poor outcome following conservative management alone. Such work to identify prognostic factors for both operative and conservative management would allow clinicians to stratify patients in terms of likely outcomes, future need for further treatment, and inform patient expectations.

## Conclusion

The current literature suggest that gastrocnemius recession is an effective treatment option for patients with plantar fasciitis who are unresponsive to conservative treatment. Gastrocnemius recession was associated with significant postoperative improvements in various foot and ankle outcome scores, ankle range of motion and power, reduction in pain, and a relatively quick return to weightbearing, work, and sports. Minor complications may occur in approximately 1/10th of patients and caution should be taken to avoid sural nerve injury, particularly when using a distal recession approach. Further research is required in the assessment of techniques to evaluate gastrocnemius contracture and identification of treatment prognostic factors.

## Supplemental Material

sj-docx-1-fai-10.1177_10711007211052290 – Supplemental material for Gastrocnemius Release in the Management of Chronic Plantar Fasciitis: A Systematic Review Click here for additional data file. Supplemental material, sj-docx-1-fai-10.1177_10711007211052290 for Gastrocnemius Release in the Management of Chronic Plantar Fasciitis: A Systematic Review by Zaki Arshad, Aiman Aslam, Mohammad A. Razzaq and Maneesh Bhatia in Foot & Ankle International

sj-pdf-2-fai-10.1177_10711007211052290 – Supplemental material for Gastrocnemius Release in the Management of Chronic Plantar Fasciitis: A Systematic Review Click here for additional data file. Supplemental material, sj-pdf-2-fai-10.1177_10711007211052290 for Gastrocnemius Release in the Management of Chronic Plantar Fasciitis: A Systematic Review by Zaki Arshad, Aiman Aslam, Mohammad A. Razzaq and Maneesh Bhatia in Foot & Ankle International
