# Supplementary material for: Gastrocnemius Release in the Management of Chronic Plantar Fasciitis: A Systematic Review
Source: Foot Ankle Int. 2021 Nov 12;43(4):568–75. doi: 10.1177/10711007211052290 (PMC8996295; doi:10.1177/10711007211052290)
Supplement: sj-docx-1-fai-10.1177_10711007211052290 – Supplemental material for Gastrocnemius Release in the Management of Chronic Plantar Fasciitis: A Systematic Review [file sj-docx-1-fai-10.1177_10711007211052290.docx]

Appendix

| Author | 1 | 2 | 3 | 4 | 5 | 6 | 7 | 8 | 9 | 10 | 11 | 12 | Total |
| --- | --- | --- | --- | --- | --- | --- | --- | --- | --- | --- | --- | --- | --- |
| Chimera et al | 2 | 0 | 2 | 2 | 0 | 1 | 2 | 0 | 2 | 0 | 2 | 2 | 15/24 |
| Hoefnagels et al | 2 | 1 | 2 | 2 | 0 | 2 | 2 | 0 | NA | NA | NA | NA | 11/16 |
| Huang et al | 2 | 2 | 1 | 2 | 0 | 2 | 2 | 0 | 2 | 2 | 2 | 2 | 19/24 |
| Monteagudo et al | 2 | 0 | 1 | 2 | 0 | 2 | 2 | 0 | 2 | 2 | 2 | 2 | 17/24 |

Online resource 1: Risk of bias and quality of evidence assessment of included case series and cohort studies using the Methodological Index for Non-Randomised Studies (MINORS) criteria. Numbers 1-12 in the first row, refer to the equivalent items in the MINORS checklist.

Online resource 2: Risk of bias and quality of evidence assessment of included randomised controlled trials using the Joanna Briggs Institute critical appraisal checklist. Numbers 1-13 in the first row, refer to the equivalent items in the Joanna Briggs Institute checklist.

| Author | 1 | 2 | 3 | 4 | 5 | 6 | 7 | 8 | 9 | 10 | 11 | 12 | 13 | Total |
| --- | --- | --- | --- | --- | --- | --- | --- | --- | --- | --- | --- | --- | --- | --- |
| Gamba et al | Y | Y | Y | NR | N | N | Y | Y | Y | Y | Y | Y | Y | 10/13 |
| Molund et al 2018 | Y | Y | Y | N | N | Y | Y | Y | Y | Y | Y | Y | Y | 11/13 |
